# Clinical evaluation of presepsin considering renal function

**DOI:** 10.1371/journal.pone.0215791

**Published:** 2019-09-06

**Authors:** Masashi Miyoshi, Yusuke Inoue, Mai Nishioka, Akishige Ikegame, Takayuki Nakao, Seiji Kishi, Toshio Doi, Kojiro Nagai

**Affiliations:** 1 Department of Medical Technology, Tokushima University Hospital, Tokushima, Japan; 2 Department of Nephrology, Tokushima University Hospital, Tokushima, Japan; Medical University of Gdansk, POLAND

## Abstract

Presepsin, a glycoprotein produced during bacterial phagocytosis, is used as a sepsis marker for bacterial infections. However, presepsin levels are affected by renal function, and the evaluation criteria according to kidney function or in chronic kidney diseases remain controversial. Furthermore, presepsin may be increased by sample stirring, but no studies have evaluated this effect.In this study, we excluded the effect of stirring by standardizing the blood collection conditions, analyzed the influence of kidney function on presepsin concentrations, and recalculated the reference range based on the findings. EDTA-whole blood from 47 healthy subjects and 85 patients with chronic kidney disease was collected to measure presepsin by PATHFAST. Presepsin was found to be significantly correlated with the levels of creatinine (r = 0.834), eGFRcreat (r = 0.837), cystatin-C (r = 0.845), and eGFRcys (r = 0.879). Furthermore, in patients with CKD, presepsin levels stratified by eGFRcys showed a significant increase in the CKD G2 patient group and with advancing glomerular filtration rate stage. The following values were obtained: Normal: 97.6 ± 27.4 pg/mL, CKD G1: 100.2 ± 27.6 pg/mL, CKD G2: 129.7 ± 40.7 pg/mL, CKD G3: 208.1 ± 70.2 pg/mL, CKD G4: 320.2 ± 170.1 pg/mL, CKD G5: 712.8 ± 336.3 pg/mL. The reference range, calculated by a nonparametric method using 67 cases of healthy volunteers and patients with chronic kidney disease G1, was found to be 59–153 pg/mL, which was notably lower than the standard reference range currently used. Presepsin concentrations were positively correlated with a few biomarkers of renal function, indicating the necessity to consider the effect of renal function in patients with renal impairment. Using the recalculated reference range considering kidney function may improve the accuracy of evaluating presepsin for diagnosis of sepsis compared to the standard reference currently in use.

## Introduction

Presepsin is a protein whose blood concentrations increase specifically during sepsis. Since its discovery in 2002 in Japan, presepsin has been widely used as a sepsis marker. Membrane-bound CD14, a surface antigen expressed on the cell membrane of monocyte macrophages and granulocytes, is a receptor for bacterial lipopolysaccharide (LPS), which activates cells via Toll-like receptor 4 [1]. Additionally, soluble CD14 present in the blood induces the activation of endothelial and epithelial cells without membrane-bound CD14 [2] and plays an important role in sensing invasion of bacteria *in vivo*. Recently, it was reported that granulocyte-mediated bacterial phagocytosis triggers elastase or cathepsin D to proteolytically cleave CD14 to produce presepsin and release it into the blood [3]. Furthermore, it was shown that the concentration of presepsin increases with infection in patients with leucopenia [4], indicating that cells other than monocytes can trigger presepsin production.

Unlike procalcitonin, which is conventionally used for sepsis diagnosis, presepsin responds very weakly to inflammation such as trauma and burn, and is considered highly specific for bacterial infection [5–7]. Compared to conventional markers, presepsin is a good clinical indicator that responds well to changes in the disease state and thus reflects the effect of therapeutics on the condition [8–10].

The presepsin cut-off value for sepsis or infectious disease diagnosis is 400–700 pg/mL (500 pg/mL: Japan) [11]. However, as reported by Nagata at el [12], presepsin levels are affected by renal function; in the blood of patients with renal disorder such as those on dialysis, the concentration can be higher, as presepsin is excreted from the kidney. Therefore, it is unsuitable to use the general cut-off values for diagnosing patients with chronic kidney disease (CKD), as the false high value associated with renal impairment can lead to erroneous judgment. Furthermore, the measured presepsin levels can be increased by sample stirring, but no studies have considered this effect, which may result in artificially high values.

In the current study, presepsin levels were measured in patients with CKD and analyzed for their relationship with the renal function index. Particularly, we focused on the relationship in early CKD stages. Additionally, by standardizing the blood collection conditions and excluding the effect of stirring, we established a reference range for evaluating the influence of kidney function.

## Materials and methods

### Design and subjects

This study enrolled 85 outpatients with CKD who visited the Tokushima University Hospital from May 2017 to September 2017. All patients were over 18 years of age and had no history of dialysis or infection symptoms. Infection was diagnosed by the physician based on apparent clinical manifestations or laboratory results. We confirmed that patients with eGFR >60 mL/min/1.73 m^2^ had abnormalities in the kidney structure such as albuminuria, urine sediment abnormalities, electrolytes, and other abnormalities due to tubular disorders or abnormalities detected by histology, structural abnormalities detected by imaging, or a history of kidney transplantation. Symptoms were present for >3 months.

Samples and data were also collected from 47 healthy volunteers without renal dysfunction as controls (Table 1).

**Table 1:** Clinical characteristics of participants in the different GFR categories.

	Normal	G1	G2	G3	G4	G5	*P* value
N	47	20	26	19	11	9	
age	30.8 ±10.5	40.6 ±11.2	56.7 ±12.5	61.8 ±14.9	66.0 ±13.3	66.9 ±13.6	< 0.01
male,n (%)	21 (44.7)	10 (50.0)	20 (76.9)	15 (78.9)	6 (54.5)	6 (66.6)	0.693
height (cm)	164.3 ±8.9	166.4 ±8.5	165.3 ±8.6	163.7 ±8.5	163.9 ±11.2	158.2 ±7.9	0.378
weight (kg)	58.7 ±11.6	69.3 ±15.6	61.9 ±11.7	67.6 ±14.9	62.7 ±13.9	55.8 ±9.5	0.032
Body surface area (m^2^)	1.634 ±0.19	1.767 ±0.21	1.501 ±0.54	1.594 ±0.50	1.677 ±0.23	1.556 ±0.14	0.217
AST (U/L)	18.0 ±6.2	23.2 ±12.7	22.9 ±8.6	24.2 ±8.0	19.1 ±6.0	21.4 ±7.6	0.420
ALT (U/L)	16.1 ±15.5	23.8 ±12.1	19.2 ±9.1	20.6 ±11.1	11.7 ±3.7	15.2 ±4.1	0.029
creatinine (mg/dL)	0.718 ±0.15	0.684 ±0.16	0.952 ±0.22	1.716 ±0.45	2.245 ±0.70	5.143 ±2.33	< 0.01
cystatine-C (mg/dL)	no data	0.710 ±0.07	1.007 ±0.11	1.657 ±0.24	2.415 ±0.33	4.021 ±0.66	< 0.01
urea nitrogen (mg/dL)	13.2 ±3.2	12.4 ±3.1	15.2 ±4.1	26.4 ±6.9	36.8 ±8.0	56.7 ±20.0	< 0.01
eGFR creat (ml/min/1.73m^2^)	93.0 ±12.9	92.4 ±18.6	63.3 ±14.3	33.0 ±9.9	23.2 ±6.2	10.2 ±3.6	< 0.01
eGFR cys (ml/min/1.73m^2^)	no data	114.2 ±14.8	73.7 ±7.2	40.1 ±7.4	23.9 ±3.7	11.3 ±3.3	< 0.01
white blood cell (*10^9^/L)	5.5 ±1.2	6.4 ±2.3	6.1 ±1.6	5.5 ±1.4	5.8 ±1.8	6.5 ±2.1	0.123
hemoglobin (g/dL)	13.6 ±1.5	14.4 ±1.6	13.8 ±1.5	13.1 ±1.6	11.7 ±1.3	10.8 ±1.4	< 0.01
hematocrit (%)	41.0 ±4.0	42.6 ±4.1	40.7 ±4.0	38.4 ±4.6	35.6 ±3.5	33.1 ±3.4	< 0.01
platelets (*10^9^/L)	247.1 ±49.4	242.1 ±64.6	245.4 ±52.1	215.3 ±43.7	353.6 ±463.9	188.2 ±43.5	0.473

Data are presented as the mean ± standard deviation, as number (%).

GFR categories: Categorized according to the KDIGO 2012 by eGFRcys.

G1: eGFRcys ≥90 mL/min/1.73 m^2^; G2: eGFRcys = 60–90 mL/min/1.73 m^2^; G3: eGFRcys = 30–60 mL/min/1.73 m^2^; G4: eGFRcys = 15–30 mL/min/1.73 m^2^; G5: eGFRcys ≤15 mL/min/1.73 m^2^.

*P* value was calculated by one-way analysis of variance.

### Blood collection and biochemical analysis

Venous blood was collected after overnight fasting. Because the presepsin concentration is increased by strong agitation, samples after complete blood count measurement cannot be used. Thus, samples were collected into EDTA-2K blood collection tubes for presepsin measurement. At the time of blood sampling, five gentle inversions were performed (S1 Fig). The blood sample was transported in a manner that prevented agitation, and the measurement was started within 10 min after blood collection.

The estimated glomerular filtration rate (eGFR) of each participant was calculated using the equation provided by the Japanese Society of Nephrology as follows:

eGFRcreat (mL/min/1.73 m^2^) = 194 × creatinine (mg/dL)^-1.094^ × Age^-0.287^ (if female, × 0.739)

eGFRcys (mL/min/1.73 m^2^) = (104 × cystatine [mg/dL]^-1.019^ × 0.996^Age^ [If female, ×0.929] - 8)

GFR was categorized according to the KDIGO 2012 by eGFRcys [13].

### Measurement of presepsin

Presepsin concentrations were measured with a compact automated immunoanalyzer “PATHFAST” (LSI Medience Corporation, Tokyo, Japan) based on a chemiluminescent enzyme immunoassay with EDTA-whole blood.

### Methods for recalculation of reference ranges

Recalculation of the reference range was performed using the nonparametric method. All values were listed in order according to the CLSI guidelines, and values of 2.5–97.5% were used as the reference range. Excision of extremes was not performed [14].

### Ethical approval

This study protocol and consent procedure were approved by the Ethics Committee of Tokushima University Hospital (No. 2699) and performed in compliance with the Helsinki Declaration. Written informed consent was obtained from all patients.

### Statistical analysis

All values are shown as the mean ± standard deviation. The results were analyzed as non-parametric variables using Mann-Whitney’s U test for comparison between two groups and using Kruskal-Wallis tests with Bonferroni *post hoc* test for multiple comparisons. Correlation was evaluated by Spearman’s correlation coefficient by a rank test. Statistical analyses were performed with EZR (Saitama Medical Center, Jichi Medical University, Saitama, Japan) [15]. A *P* value < 0.05 was considered to indicate significance.

## Results

### Comparison between normal and chronic renal failure

**Fig 1:**
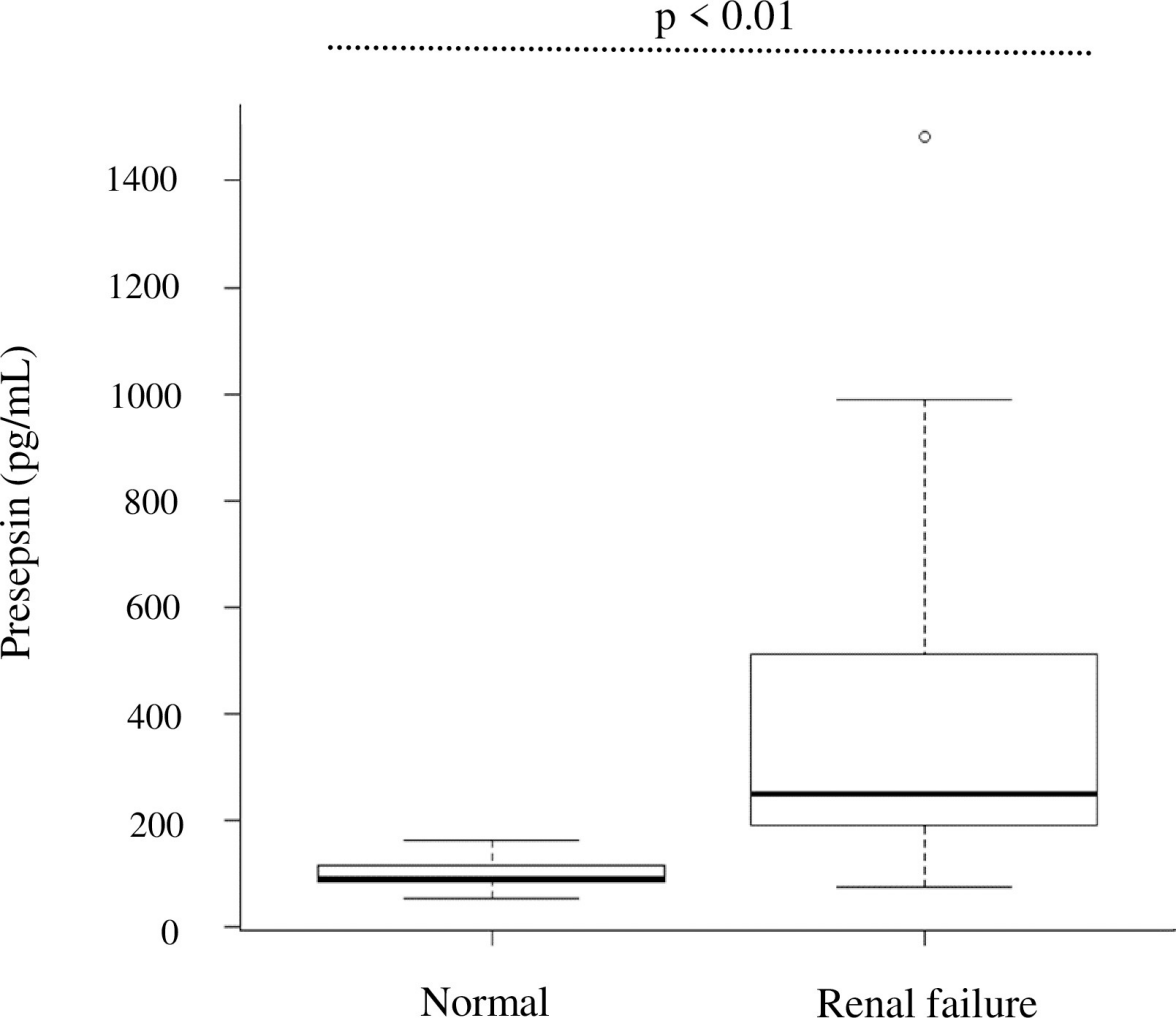
Presepsin levels in control and renal failure groups. Renal failure group: CKD G4+ G5.*P* value was calculated using the Mann–Whitney U test.

The presepsin levels in the chronic renal failure group (CKD G4 · G5) was significantly higher (410.1 ± 318.9 pg/mL) than that in the normal group (97.6 ± 27.4 pg/mL) (*P* = 0.01) (Fig 1).

### Correlation with renal functional indices

**Fig 2:**
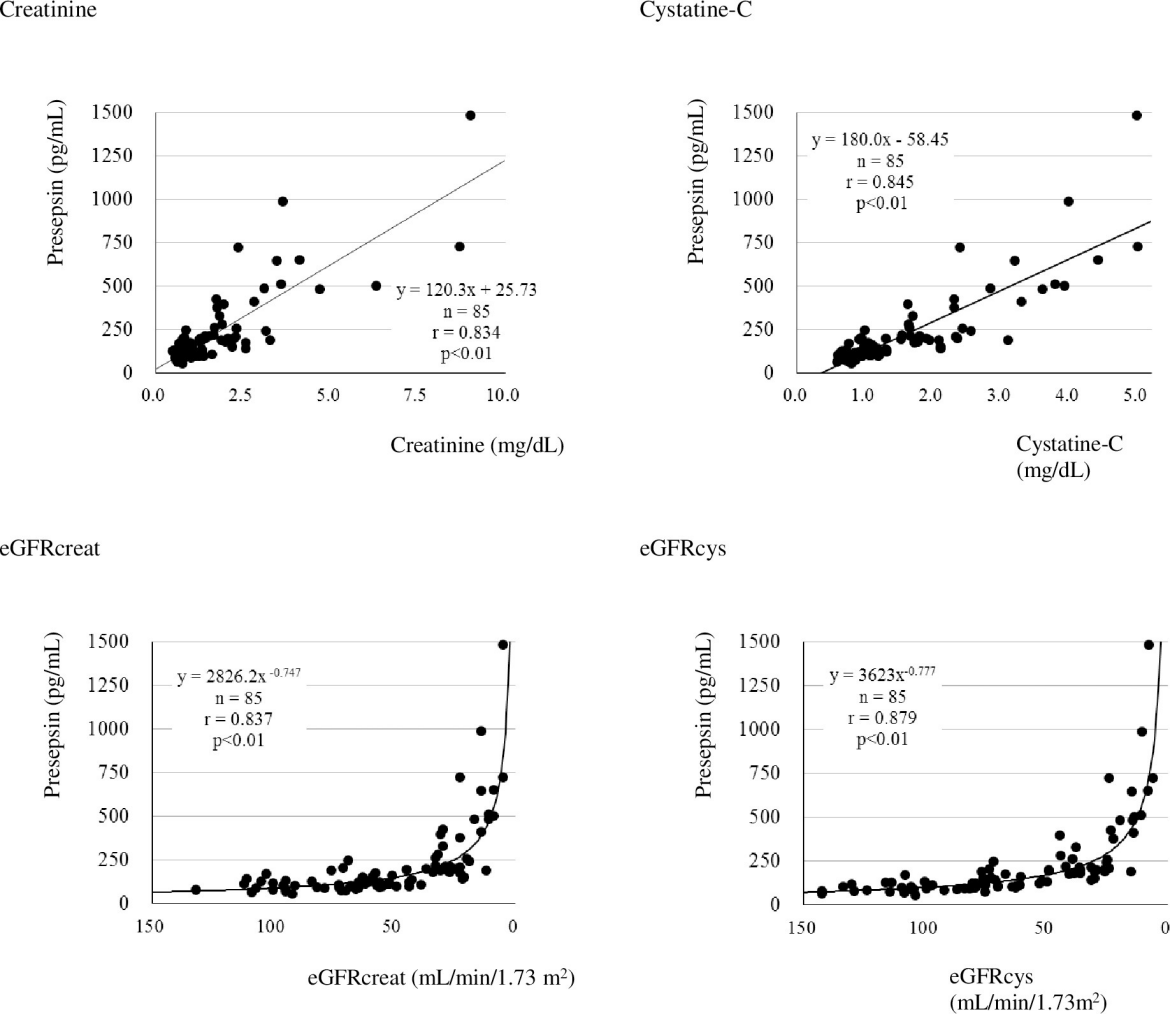
Correlation between presepsin values and renal function index. *P* value was calculated with Spearman’s correlation coefficient test.eGFRcreat (mL/min/1.73 m^2^) = 194 × creatinine (mg/dL)^-1.094^ × Age^-0.287^ (if female, × 0.739) eGFRcys (mL/min/1.73 m^2^) = (104 × cystatine [mg/dL]^-1.019^ × 0.996^Age^ [If female, ×0.929]^-8^).

The presepsin levels were analyzed for their correlation with creatinine, eGFRcreat, cystatin-C, and eGFRcys. The correlation coefficients were as follows: creatinine, r = 0.834; eGFRcreat, r = 0.837; cystatin-C, r = 0.845; and eGFRcys, r = 0.879. All correlations were significant, and presepsin levels increased with deterioration of renal function (Fig 2).

### Stratified comparison in CKD patients

**Fig 3:**
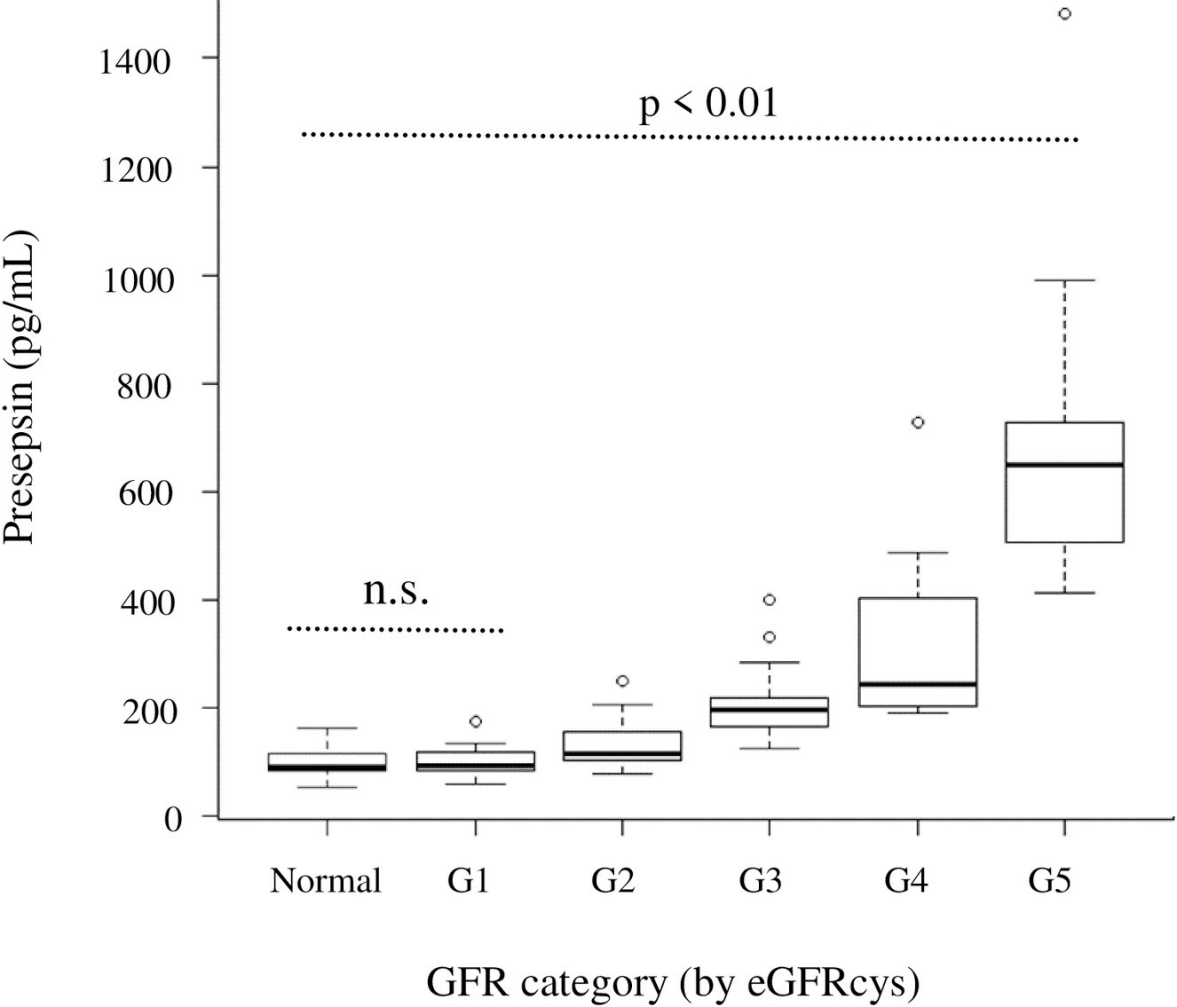
Plot of presepsin concentrations in controls or patients with chronic kidney disease vs GFR stage classified by eGFRcys. *P* value was calculated by Kruskal-Wallis one-way analysis of variance. *P* value adjustment was calculated using the Bonferroni method.

In patients with CKD, presepsin levels were stratified by GFR stage classified by eGFRcys (Fig 3). The presepsin levels in each group were: Normal: 97.6 ± 27.4 pg/mL, CKD G1: 100.2 ± 27.6 pg/mL, CKD G2: 129.7 ± 40.7 pg/mL, CKD G3: 208.1 ± 70.2 pg/mL, CKD G4: 320.2 ± 170.1 pg/mL, CKD G5: 712.8 ± 336.3 pg/mL.

As a result, presepsin levels showed a significant tendency to increase in the CKD G2 patient group, which is regarded as the mildly declining group, as the GFR stage increased. In contrast, in patients with CKD G1, in which renal function was maintained, there was no significant difference between presepsin levels in patients and control individuals.

### Recalculation of reference range considering renal function

A total of 67 cases in the normal group and CKD G1 patient group were used to determine the reference range calculated using a nonparametric method. The recalculated reference range was 59–153 pg/mL, which was lower than the reference range of 98.3–314 pg/mL provided by the reagent manufacturer of PATHFAST.

## Discussion

Presepsin levels are affected by altered renal function and sample agitation. However, in previous studies, the effects of agitation were not considered, and the possibility of a false high level could not be excluded.

This study, which eliminated the influence of stirring and sampling, clarified that presepsin concentrations are strongly correlated with various renal function indices and tended to increase with renal function deterioration.

The molecular weight of presepsin is approximately 13 kDa, which is similar to that of cystatin-C. Presepsin levels increase and accumulate in the blood when renal function is impaired in patients with renal disorders.

As significant correlation was found for all indices examined in this study. Interestingly, eGFRcreat, which is generally used as a renal function index, rather than creatinine, showed the same correlation as creatinine. Because the presepsin level was not dependent on the individual’s sex or age, these factors were predicted to have no significant influence on the presepsin value. In the stratified comparison, a significant difference was found in both eGFRcreat and eGFRcys. However, for eGFRcys, the influence from a lower range was confirmed (S2 Fig). This was likely because cystatin C reflected kidney disorder from an earlier stage compared to creatinine. We corrected the sampling to measure presepsin and analyzed the data by using eGFRcys. Therefore, a significant false high value reflecting renal function was confirmed in a group of patients with early-stage CKD compared as that found by Nagata et al by using eGFRcreat.

Although presepsin showed an increasing trend in the early stage due to the deterioration of renal function, there was no significant difference between healthy subjects and patients in the CKD G1 group. Thus, the CKD G1 group may be diagnosed as normal. In patients in the CKD G2 group and higher stages, a significant increase in presepsin was observed even in uninfected cases. Sepsis diagnosis is not based on only one biomarker, but is a comprehensive diagnosis based on multiple markers and clinical symptoms. Presepsin levels must be carefully interpreted to diagnosis sepsis in patients with renal impairment.

Although it has been reported that presepsin is related to age and hemoglobin levels, multivariate analysis using these factors as explanatory variables revealed no independent causality other than renal function (S1 Table). It is possible that renal clearance was decreased by the influence of aging or renal anemia may have influenced presepsin levels, but no direct causal relationship was observed.

Additionally, false high values of presepsin arising due to vigorous agitation of the blood samples have been reported, preventing accurate clinical assessment. Mechanical stimuli such as agitation lead to the formation of macromolecular complexes, which reacts with the anti-presepsin antibody in the reagent in a larger molecular weight fraction. Although the false high value is considered to be due to cross-reaction with this polymer complex, the mechanism of complex formation has not been clarified. There are no efficient methods other than avoiding agitation of the sample at the time of blood collection/delivery to prevent these effects, necessitating extreme caution. Although doctors and nurses collect blood at the bedside with caution, fluctuations at the pre-measurement stage can be large, affecting the reliability of the results. Therefore, it is essential to educate clinical practitioners regarding specimen collection.

In this study, recalculation of the reference range using data from the CKD G1 group and normal group indicated remarkably low values 59–153 pg/mL, which were half of the reference range currently in use. The sample group used to calculate the reference range by the reagent manufacturer did not consider the effects of renal function or agitation, and it is likely that these false high values were included. Therefore, this sample group may not be appropriate for calculating the reference range. In contrast, the sample group used for recalculation in this study excluded false high value cases caused by renal function and agitation, making it a more useful index.

The new reference range obtained in this study can be used for the early diagnosis of sepsis in patients with normal kidney function. However, the new reference range obtained in this study did not meet the requirement for 120 cases recommended by CLSI guidelines and may not be sufficient evidence for establishing a new reference range [14]. Further larger studies are needed to establish a more reliable reference range.

This study had some limitations. First, the presence or absence of an infectious disease could not be completely excluded, as this factor was based on the judgment of the physician. Second, in the sample group examined in this study, there was an age difference between the normal group and CKD patient group. Although age was not an independent variable, normal groups with no difference in age should be considered as the control group.

In conclusion, by excluding the effect of stirring during sampling, our study demonstrated that presepsin levels exhibit an increasing trend as GFR decreases. Currently, the false high values obtained using the standard reference range are largely ignored. Therefore, using the newly recalculated reference range, which excludes the effects of renal function and agitation, may improve the efficiency in sepsis diagnosis, particularly in the early stage. However, we recommend larger studies to confirm these findings and support alternate reference ranges.

## Supporting information

S1 Fig Effect of agitation on presepsin measurements. *P* value was calculated using paired *t* test.
(PDF)

S2 Fig Plot of presepsin concentrations in patients with chronic kidney disease vs GFR stage classified by eGFRcreat. *P* value was calculated by Kruskal-Wallis one-way analysis of variance.
*P* value adjustment was calculated using the Bonferroni method.
(PDF)

S1 Table Univariable analysis and multivariate linear regression analysis of characteristics relative to presepsin in patients with chronic kidney disease. * Pearson`s correlation coefficients.
(PDF)

## References

[1] Wright SD, Ramos RA, Tobias PS, Ulevitch RJ, Mathison JC. CD14, a receptor for complexes of lipopolysaccharide (LPS) and LPS binding protein. Science 1990;249: 1431–1433. 10.1126/science.1698311 1698311

[2] Tapping RI, Tobias PS. Cellular binding of soluble CD14 requires lipopolysaccharide (LPS) and LPS binding protein. J Biol Chem 1997;272: 23157–23164. 10.1074/jbc.272.37.23157 9287319

[3] Arai Y, Mizugishi K, Nonomura K, Naitoh K, Takaori-Kondo A, Yamashita K. Phagocytosis by human monocytes is required for the secretion of presepsin. J Infect Chemother 2015;21: 564–569. 10.1016/j.jiac.2015.04.011 26026662

[4] Koizumi Y, Shimizu K, Shigeta M, Okuno T, Minamiguchi H, Kito K, et al Plasma presepsin level is an early diagnostic marker of severe febrile neutropenia in hematologic malignancy patients. BMC Infect Dis 2017;17: 27 10.1186/s12879-016-2116-8 28056845 PMC5217328

[5] Henriquez-Camacho C, Losa J. Biomarkers for sepsis. BioMed Res Int 2014;2014: 547818 10.1155/2014/547818 24800240 PMC3985161

[6] Castelli GP, Pognani C, Meisner M, Stuani A, Bellomi D, Sgarbi L. Procalcitonin and C-reactive protein during systemic inflammatory response syndrome, sepsis and organ dysfunction. Crit Care 2004;8: 234–242. 10.1186/cc2875 15312223 PMC522844

[7] Dupuy AM, Philippart F, Péan Y, Lasocki S, Charles P-E, Chalumeau M, et al Role of biomarkers in the management of antibiotic therapy: an expert panel review: I—currently available biomarkers for clinical use in acute infections. Ann Intensive Care 2013;3: 22 10.1186/2110-5820-3-22 23837559 PMC3708786

[8] Yaegashi Y, Shirakawa K, Sato N, Suzuki Y, Kojika M, Imai S, et al Evaluation of a newly identified soluble CD14 subtype as a marker for sepsis. J Infect Chemother 2005;11: 234–238. 10.1007/s10156-005-0400-4 16258819

[9] Endo S, Suzuki Y, Takahashi G, Shozushima T, Ishikura H, Murai A, et al Presepsin as a powerful monitoring tool for the prognosis and treatment of sepsis: A multicenter prospective study. J Infect Chemother 2014;20: 30–34. 10.1016/j.jiac.2013.07.005 24462421

[10] Endo S, Suzuki Y, Takahashi G, Shozushima T, Ishikura H, Murai A, et al Usefulness of presepsin in the diagnosis of septic in a multicenter prospective study. J Infect Chemother 2012;18: 891–897. 10.1007/s10156-012-0435-2 22692596

[11] Wu CC, Lan HM, Han ST, Chaou CH, Yeh CF, Liu SH, et al Comparison of diagnostic accuracy in sepsis between presepsin, procalcitonin, and C-reactive protein: a systematic review and meta-analysis. Ann Intensive Care 2017;7: 91 10.1186/s13613-017-0316-z 28875483 PMC5585118

[12] Nagata T, Yasuda Y, Ando M, Abe T, Katsuno T, Kato S, et al Clinical impact of kidney function on presepsin levels. PLoS One 2015;10: e0129159 10.1371/journal.pone.0129159 26030716 PMC4451771

[13] KDIGO work group. KDIGO 2012 clinical practice guideline for the evaluation and management of chronic kidney disease. Kidney Int Suppl 2013;3: 1–150. 10.1038/ki.2013.243 23989362

[14] Wayen PA. CLSI C28-A3: Defining, establishing, and verifying reference intervals in the clinical laboratory; approved guideline -third edition. Clinical and Laboratory Standards Institute 2008.

[15] Kanda Y. Investigation of the freely available easy-to-use software ‘EZR’ for medical statistics. Bone Marrow Transplant 2013;48: 452–458. 10.1038/bmt.2012.244 23208313 PMC3590441

