# Supplementary figures and images for: Clinical evaluation of presepsin considering renal function
Source: PLoS One. 2019 Sep 6;14(9):e0215791. doi: 10.1371/journal.pone.0215791 (PMC6730850; doi:10.1371/journal.pone.0215791)

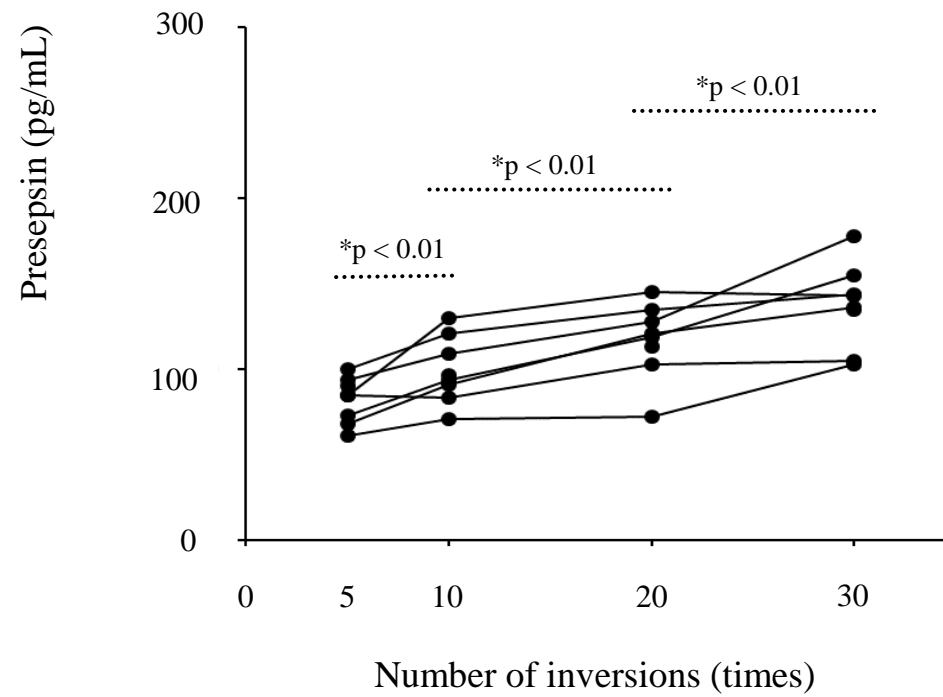

S1 Fig. Effect of agitation on prepsin measurement.

Supplement: S1 Fig — P value was calculated using paired t test. (PDF) [file pone.0215791.s001.pdf]
