# Supplementary material for: Clinical evaluation of presepsin considering renal function
Source: PLoS One. 2019 Sep 6;14(9):e0215791. doi: 10.1371/journal.pone.0215791 (PMC6730850; doi:10.1371/journal.pone.0215791)
Supplement: S2 Fig — P value was calculated by Kruskal-Wallis one-way analysis of variance. P value adjustment was calculated using the Bonferroni method. (PDF) [file pone.0215791.s002.pdf]

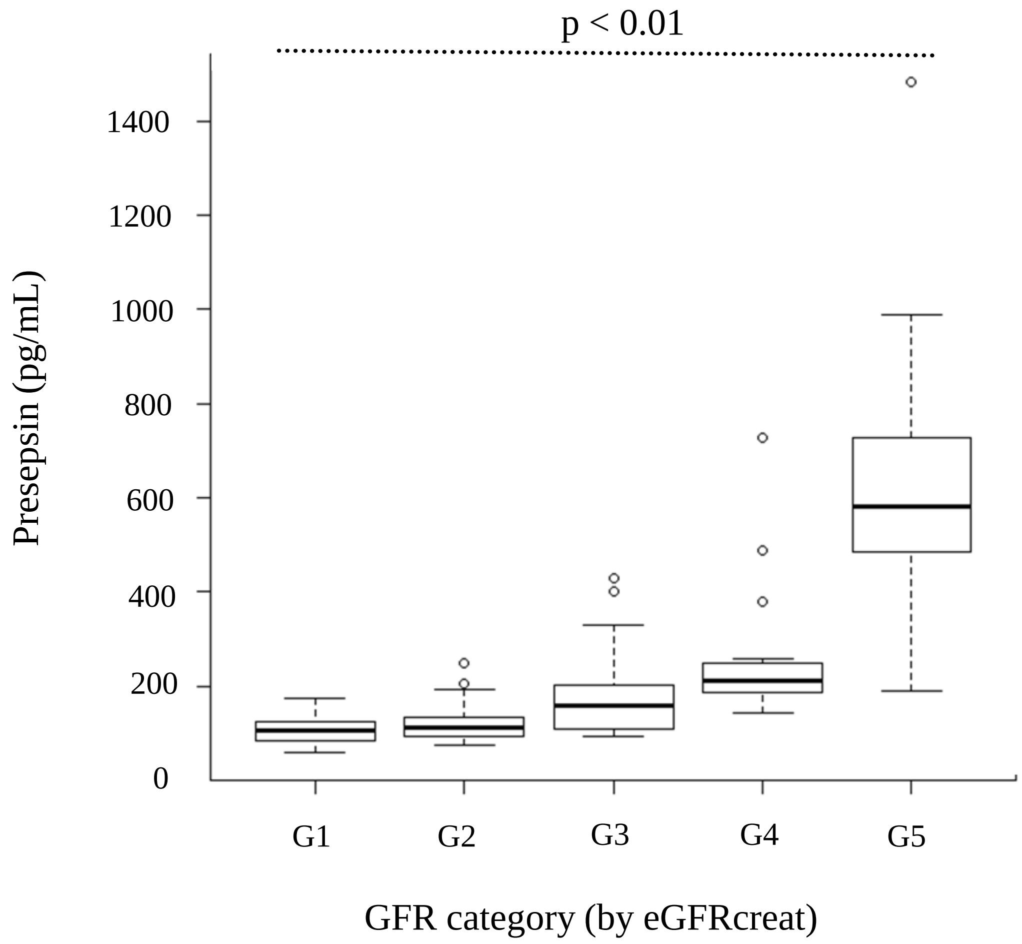

S2 Fig. Plot of presepsin concentrations in patients with chronic kidney disease vs GFR stage classified by eGFRcreat.
