# Supplementary material for: Clinical evaluation of presepsin considering renal function
Source: PLoS One. 2019 Sep 6;14(9):e0215791. doi: 10.1371/journal.pone.0215791 (PMC6730850; doi:10.1371/journal.pone.0215791)
Supplement: S1 Table — * Pearson`s correlation coefficients. (PDF) [file pone.0215791.s003.pdf]

S1 Table. Univariable analysis and multivariate linear regression analysis of characteristics relative to presepsin in patients with chronic kidney disease.

	Univariable		Multivariate	
	Correlation coefficient	*P* value	Estimate	*P* value
age	0.355	< 0.01 *	0.978	0.253
male,n (%)	-0.093	0.395	-	-
Body surface area (m^2)	-0.138	0.216	-	-
AST (U/L)	0.102	0.399	-	-
ALT (U/L)	-0.085	0.485	-	-
urea nitrogen (mg/dL)	0.645	< 0.01 *	-7.247	< 0.01
cystatine-C (mg/dL)	0.845	< 0.01 *	270.84	< 0.01
white blood cell ($*10^9$/L)	0.130	0.237	-	-
hemoglobin (g/dL)	-0.522	< 0.01 *	-1.576	0.845
hematocrit (%)	-0.525	< 0.01 *	-	-
platelets ($*10^9$/L)	0.148	0.177	-	-

(Adjusted R-squared : 0.7588)
